# Molecular autopsy by proxy: relevance for genetic counseling in rare genetic disorders

**DOI:** 10.3389/fgene.2024.1400295

**Published:** 2024-05-27

**Authors:** Cristina Skrypnyk, Rawan AlHarmi

**Affiliations:** ^1^ Assistant Professor, Molecular Genetics, Princess Al-Jawhara Al-Ibrahim Center for Molecular Medicine, Genetics, and Inherited Disorders and Molecular Medicine Department, College of Medicine and Medical Sciences, Arabian Gulf University, Manama, Bahrain; ^2^ Consultant Medical Geneticist, University Medical Clinics, Manama, Bahrain; ^3^ Research Associate, Regenerative Medicine Unit, Arabian Gulf University, Manama, Bahrain

**Keywords:** molecular autopsy by proxy, genetic counseling, genetic testing, carrier testing, exome sequencing, autosomal recessive disorders

## Abstract

**Background:**

Rare genetic disorders may result in death before a definitive clinical diagnosis is established.

**Aim:**

This study aims to outline the processes and challenges in managing, from a genetic perspective, couples who lost children affected by rare genetic disorders.

**Results:**

Six couples who experienced child loss due to rare genetic disorders, seen by the primary author at genetic evaluation and counseling sessions, were retrospectively analyzed. Four out of 6 couples reported consanguinity. Exome and genome sequencing were performed for the parents. Carrier status of two rare lethal metabolic disorders was confirmed in one consanguineous couple. Three couples were carriers of 3 other rare diseases. Variants of LYST, MPV17, HEXB, ITGB4, CD3E, ASPM, TK2, COL11A2, and LAMB3 genes were identified. Six out of 10 were pathogenic variants, out of which 4 correlated with the demised children’s phenotypes. One couple was negative for pathogenic variants. The last couple did not undergo genetic testing since they were beyond the fertile window.

**Conclusion:**

Appropriate parental genetic evaluation and counseling are mandatory for selecting the right genetic test to certify the diagnosis *postmortem*, by virtue of molecular autopsy by proxy. Clarifying a rare disorder diagnosis can help couples to avoid recurrence and plan early for their next pregnancies.

## Introduction

Genomic era is facilitating a faster diagnosis of rare genetic disorders with complex phenotypes. Some of these disorders may result in death prior to the clinical diagnosis being fully confirmed through genetic testing. Delayed diagnosis can lead to even more children affected and deceased. The majority of such lethal disorders which are not diagnosed and missed prior to the child’s death are recessive. Consanguinity is known to contribute to genetic recessive disorders in particular (Shawky et al., 2013).

Molecular autopsy or *postmortem* genetic testing examines genetic factors as causes of death (Koike et al., 2022). It is a tool that can be added to a set of tests conducted during *postmortem* evaluation (Edwards, 2005). In forensic medicine, molecular autopsy is performed to ascertain the cause of death in negative autopsies. Such cases are encountered usually in young decedents in whom an underlying inherited arrhythmogenic syndrome is the main suspected cause of death (Martínez-Barrios et al., 2023). Samples collected include blood and fresh or frozen tissue (heart, liver, muscle or spleen tissue) (Sanchez et al., 2016; Stiles et al., 2021). Molecular autopsy was proved efficient in solving complex cases since more than 20 years (Ackerman, Tester, and Driscoll, 2001). Spectrums of conditions where molecular autopsy is employed include sudden unexplained death syndrome (SUDS), sudden infant death syndrome (SIDS), and sudden unexpected death in epilepsy (SUDEP) (Ackerman et al., 2011; Fialho et al., 2021). Additionally, pharmacogenomics can be used to determine drug-related causes and/or the manner of death (Wendt and Budowle, 2020).

Molecular autopsy by proxy (MABP) is a term used to describe the genetic analysis conducted on family members of deceased individuals with the purpose of determining the underlying cause of their death (Alghamdi et al., 2021). Knowledge of the phenotype and pathology with the subsequent selection of the appropriate test can save time and other lives. Technologies used in molecular autopsy and MABP include Sanger sequencing, next-generation sequencing (NGS) as personalized panels, exome sequencing (ES), and even genome sequencing (GS) (Martínez-Barrios et al., 2023). The diagnostic rate of ES has been demonstrated to be significantly high. Employing ES for MABP is practical and acceptable for the families, particularly in the Middle East, with an excellent yield of diagnosis clarification (Shamseldin et al., 2018).

The aim of this study is to outline the processes and challenges in managing, from a genetic perspective, couples who lost children affected by rare genetic disorders.

## Materials and methods

### Study design

This is a retrospective analysis of couples seen by the primary author for genetic evaluation and counseling at the University Medical Clinics, King Abdullah Medical City, Arabian Gulf University, Bahrain, who lost children affected by rare genetic disorders between 2011 and 2023. Data were collected from the medical records, reviewing the reason for referral, family history of genetic disorders, available medical reports, available biological specimens from the deceased/affected children, the genetic testing strategy, and its outcomes.

### Human subjects

Six couples were included in the study.

### Genetic testing

ES and GS were carried out in collaboration with external genetic diagnostic laboratories.

### Ethical considerations

Ethical approval was obtained from the Research and Ethics Committee of the Arabian Gulf University (Serial number: E33-PI-02-24). A written informed consent was obtained from each of the couples, also serving as the child’s legal guardian/next of kin.

## Results

Six clinically asymptomatic couples who experienced the loss of children presumed to be affected by rare genetic disorders were included in the study. The age range of mothers was 24–52 years and that of fathers was 31–54 years. Four out of 6 couples reported consanguinity. Specific genetic tests were recommended to the couples with a special consideration of the most suspected disorders. A biological sample was available from a child of one couple only. The last couple did not undergo genetic testing since they were out of the fertile window at the time of consultation. Parental ES and GS were negative for pathogenic variants in one couple. Details of the included couples and their children are shown in Table 1.

**TABLE 1:** Details of the clinical and genetic findings of the study cases.

Couple	Couple I	Couple II	Couple III	Couple IV	Couple V	Couple VI
Parental age	Mother 24 years old; Father 38 years old	Mother 38 years old; Father 36 years old	Mother 31 years old; Father 37 years old	Mother 28 years old; Father 38 years old	Mother 29 years old; Father 31 years old	Mother 52 years old; Father 54 years old
Consanguinity	First-degree cousins	First-degree cousins	First-degree double cousins	Non-consanguineous	Non-consanguineous	Second-degree cousins
Reason for consultation	Two miscarriages in the 1st trimester; two children died in infancy	Four miscarriages in the 1st trimester; one child died in infancy	One miscarriage; one child died as a newborn	Two children died as newborns	Two children with a similar phenotype died at the age of 1 year	Four children with a similar phenotype died in childhood
Referral by a specialist	No	Yes	Yes	No	No	Yes
Prior clinical diagnosis (Affected child/children)	CHS (1st child) MDDS (2nd child)	Metabolic disorder; suspicion of Leukodystrophy	EBS	EB; junctional or dystrophic type (unknown)	Plurimalformative syndrome	MPS III (Sanfilippo syndrome)
Prior medical reports	Available	Available	Available	Available	Available	Incomplete
Prior genetic testing	1st child not tested; 2nd child tested negative by DGUOK gene MLPA	No	Child tested negative by COL7A1 gene MLPA	No	1st child not tested; 2nd child tested negative by array and ERCC6 gene sequencing	No
Years without a certified diagnosis	2	5	5	4	7	8
Family history	Strongly positive for clinical and pathological diagnosis of CHS	Positive for death in infancy	Positive for Stickler syndrome	Negative	Negative	Negative
Clinical history (Affected child/children)	1st child phenotype: fever, lower respiratory tract infections, white blood cell giant granules, thrombocytopenia, and hepatomegaly. 2nd child phenotype: prolonged neonatal jaundice and hepatomegaly	Global developmental delay, macrocephaly, severe hypotonia, brain MRI with extensive white matter and basal ganglia changes with cerebellar hypoplasia, and low leucocyte levels of total hexosaminidase	Polyhydramnios. Differential diagnoses of EB types considered	Blistering that affected their fingers, toes, and body extremities, generalized and fast leading to death in the 1st month of life	IUGR, oligohydramnios, premature birth at 36 weeks, severe microcephaly, narrowing of the bitemporal diameter, low hair line insertion, depressed nasal bridge, long eyelashes, long philtrum, micrognathia, big cheeks, large hiatal hernia, severe gastroesophageal reflux, generalized hypertonia with joint contractures, brain MRI with marked atrophic changes and significant callosal thinning and delayed white matter myelination, atrial septal defect with shunting, severe respiratory distress that required intubation	Fitting with the prior clinical diagnosis of MPS III
Diagnosis after consultation	Two rare metabolic disorders running in the same family. Differential diagnosis of variable forms of CHS and MDDS were considered	Suggestive of lysosomal lipid storage disease. Suspicion of the severe infantile form of Sandhoff disease	Lethal type of non-Herlitz JEB	Lethal type of JEB-H	Differentials include: Microdeletion or microduplication (del1q42, del6q or dup4q), Cockayne syndrome II, Pena-Shokeir syndrome II, and Schinzel-Giedion syndrome	Possibility of MPS III
Biological sample available (Affected child/children)	No	No	No	No	Available from the 2nd but not the 1st child	No
Genetic testing recommended (Pretesting counseling)	ES DUO* (mother and father) with filters for metabolic and mitochondrial nuclear disorders	ES DUO* (mother and father) with filters for metabolic disorders	ES DUO** (mother and father) with filters for skin disorders (EB) and connective tissue disorders	ES DUO* (mother and father) with filters for skin disorders (EB as a priority)	ES TRIO*** (2nd child, mother, and father) with filters for polymalformative syndromes	ES DUO (mother and father) with filters for metabolic disorders
Diagnosis after testing	Couple are carriers of two rare lethal metabolic disorders: CHS and MDDS	Couple are carriers of Severe infantile GM2 gangliosidosis (Sandhoff disease)	Couple are carriers of JEB5B (Carmi syndrome)	Couple are carriers of a severe and lethal form of JEB-H	ES and GS reported negative	Genetic testing not performed as the couple are beyond the conception window
Post-testing counseling	25% recurrence risk for each of these rare metabolic disorders. PGT-M recommended	25% recurrence risk for GM2 gangliosidosis. PGT-M recommended	25% recurrence risk for JEB5B. PGT-M recommended	25% recurrence risk for JEB-H. PGT-M highly recommended	No final diagnosis was reached. 25% recurrence risk. A *de novo* variant and parental germline mosaicism must be considered with a recurrence risk of 1%–4%. PGT-M is not feasible	Relief of understanding the mechanisms of the disorder and the inheritance pattern
Follow up	PGT-M	PGT-M	PGT-M	PGT-M	Natural pregnancy with a normal child	No further action

CHS, Chédiak-Higashi syndrome; COL7A1, Collagen type VII, alpha-1 chain; DGUOK, Deoxyguanosine kinase; EB, Epidermolysis bullosa; EBS, Epidermolysis bullosa simplex; ERCC6, Excision repair cross-complementing group 6; IUGR, Intrauterine growth restriction; JEB, Junctional epidermolysis bullosa; JEB-H, Epidermolysis bullosa, junctional, Herlitz type; JEB5B, Epidermolysis bullosa, junctional 5B, with pyloric atresia; MDDS, Mitochondrial DNA, depletion syndrome; MLPA, Multiplex ligation-dependent probe amplification; MPS, Mucopolysaccharidosis; MRI, Magnetic resonance imaging; PGT-M, Preimplantation genetic testing for monogenic disorders; ES, Exome sequencing; GS, Genome sequencing; *Genome Diagnostics, Nijmegen, The Netherlands; **Saudi Diagnostic Limited Company, Riyadh, Saudi Arabia; ***Centogene, Rostock, Germany.

Common carrier status of LYST and MPV17 gene variants linked to Chédiak-Higashi syndrome (CHS) and mitochondrial DNA depletion syndrome (MDDS), respectively, were found in a consanguineous couple (couple I). Common carrier status of HEXB, ITGB4, and LAMB3 gene variants were identified in the other couples, linked to severe infantile GM2 gangliosidosis (Sandhoff disease) (couple II), Epidermolysis Bullosa, Junctional 5B, with pyloric atresia (JEB5B) (couple III), and Epidermolysis bullosa junctional Herlitz type (JEB-H) (couple IV), respectively. Furthermore, in couple IV, the mother was identified as a carrier of 4 other variants of CD3E, ASPM, TK2, and COL11A2 genes. In total, 4 out of the 6 pathogenic identified variants correlated with the phenotype of the demised children and were predicted as being deleterious by the Combined Annotation Dependent Depletion (CADD) score, with a scaled score ranging from 23.2 to 35 (https://cadd.gs.washington.edu/snv). Carrier status of the parents correlated with the full phenotype of these disorders in their affected children and confirmed their diagnoses *postmortem*. ES achieved the molecular diagnosis in 8 out of 11 tested individuals, reaching a diagnostic yield of approximately 73%. Additionally, this explained the presence of some of these disorders in their extended families. Table 2 demonstrates the genetic variants identified among the families.

**TABLE 2:** Genetic variants identified among the study cases.

Couple	Parent	Gene	Genetic variant	Zygosity	Novelty	Classification	CADD score
Couple I	Mother	MPV17	c.278A>C (p.Gln93Pro)	Het	Reported in ClinVar and VarSome	P/LP	32
		LYST	c.2311C>T (p.Gln771*)		ClinVar	LP/VOUS	37
		LYST	c.146G>A (p.Gly49Asp)		ClinVar	VOUS	26.2
	Father	MPV17	c.278A>C (p.Gln93Pro)	Het	ClinVar and VarSome	P/LP	32
		LYST	c.2311C>T (p.Gln771*)		ClinVar	LP/VOUS	37
Couple II	Mother	HEXB	c.1238_1242del (p.Lys414Cysfs*7)	Het	ClinVar and VarSome	P	34
	Father						
Couple III	Mother	ITGB4	c.1234dupC (p.Leu412fs)	Het	VarSome	P	23.2
		CD3E	c.103 + 1G>A		ClinVar and VarSome	LP	23.1
		ASPM	c.4363G>T (p.E1455)		ClinVar	P	35
		TK2	c.441del (p.Y148lfs*12)		ClinVar	LP	10.19
		COL11A2	c.2081_2085delinsA (p.Gly694fs)		ClinVar and VarSome	P/LP	25.3
	Father	ITGB4	c.1234dupC (p.Leu412fs)	Het	VarSome	P	23.2
Couple IV	Mother	LAMB3	c.958_1034dup (p.Asn345Lysfs*77)	Het	ClinVar and VarSome	P	29.6
	Father						

ASPM, Abnormal spindle-like, microcephaly-associated; CADD, Combined annotation dependent depletion (https://cadd.gs.washington.edu/snv); CD3E, CD3epsilon; ClinVar, (https://www.ncbi.nlm.nih.gov/clinvar/); COL11A2, Collagen type XI alpha-2 chain; Het, heterozygous; HEXB, Hexosaminidase subunit beta; ITGB4, Integrin subunit beta 4; LAMB3, Laminin subunit beta-3; LP, Likely pathogenic; LYST, Lysosomal trafficking regulator; MPV17, Mitochondrial inner membrane protein; P. Pathogenic; TK2, Thymidine kinase 2; VarSome, (https://varsome.com); VOUS, Variant of uncertain clinical significance.

## Discussion

Couples may seek genetic counseling following the demise of a child. Despite the presence of highly advanced technologies for the diagnosis of genetic disorders, detailed history taking, clinical phenotyping, and the proper use of medical records facilitate and guide the decision and choice of the genetic test. The success of *postmortem* genetic testing requires a multidisciplinary team of laboratory geneticists, molecular pathologists, clinical geneticists, and genetic counselors (Deignan et al., 2023). We present, in a detailed approach, a series of couples who visited the clinic for counseling following miscarriage or child demise with suspected genetic disorders with the aim to understand the cause, the recurrence risk, and be advised on interventions to avoid future recurrence.

Consultations need to be comprehensive and empathetic in order to understand involved mechanisms and relevant risk factors. Genetic counseling shall be carried out respecting the autonomy, life principles, and moral, religious, and cultural values of the consultands. Medical reports are essential for adequate assessment of past medical history and were mostly available and helped in the assessment and decision making. A preliminary clinical diagnosis of a genetic disorder was suspected in the deceased children but genetic testing either was not conducted or did not provide confirmation. Consultands in our series needed clear and repeated explanations of the mechanisms of inheritance and particularities of the rare genetic disorders identified. Moreover, recurrent miscarriages and positive family history were not particularly emphasized by the couples as they did not identify connections with the demise of their children. As reported in other studies, parents need time to absorb the genetic testing results (Liang et al., 2022). Similarly, more than one visit was needed for the parents in our cohort to grasp the information provided and ask questions. The initial visit was essential to establish rapport. In the next visits, it was important to clarify misconceptions, discuss the implications of the diagnosis to other family members, and the couples were encouraged to share this information with their relatives. For parents with alive children with a genetic disorder, contact information of other patients with the same condition and advocacy groups can be provided by the genetic counselor for support (Hartley et al., 2011).

Double carrier status of CHS and MDDS was identified in couple I and correlated with the previous clinical diagnoses of their lost children. MDDS has a complex heterogeneity, therefore ES is superior to targeted gene sequencing approach, previously employed for one of the lost children of this couple. Severe infantile GM2 gangliosidosis (Sandhoff disease) (couple II), JEB5B (couple III), and JEB-H (couple IV) were confirmed and correlated with the clinical, biochemical, and radiological diagnoses of the affected children. Furthermore, in couple III, it was determined that the mother, who has a positive family history of Stickler syndrome, carries a pathogenic variant in the COL11A2 gene. Couple VI, suspected to be carriers of mucopolysaccharidosis (MPS) III, were not tested as they were beyond the window of conception at the time of presentation.

MPS III, gangliosidosis, and mitochondrial disorders have been reported in Bahrain (Al-Arrayed, 2012). In a retrospective study conducted in the largest hospital in Bahrain, including data of 17 years from 2000 to 2017, MPS III and MDDS were reported. Two patients were diagnosed with MDDS type 6 with a homozygous variant in MPV17 gene [c.278A>C (p.Gln93Pro)], which is the exact same variant detected in couple I from our cohort. GM1 but not GM2 gangliosidosis was reported in the study (Aljishi et al., 2019). No data could be found in the literature on JEB5B and JEB-H in Bahrain.

Studies utilizing molecular autopsy and MABP are scarce in the region. Such studies are essential to ascertain whether the demise of the child can be explained by genetic disorder and to implement earlier diagnostic and therapeutic strategies (Owen et al., 2023). Three similar studies were conducted in Saudi Arabia, where consanguineous marriages are customary (Shamseldin et al., 2018; Alghamdi et al., 2021; Shamseldin et al., 2021). However, of the gene variants detected in our cohort, only a variant of COL11A2 was reported in a child with micromelia and craniofacial dysmorphia (Shamseldin et al., 2018). Nevertheless, authors from other regions of the world have reported variants of the same genes identified in our cohort (Webster et al., 2021). Reported the presence of a LAMB3 gene variant in the mother of a SUDS case. A different LYST variant was reported by (Halvorsen et al., 2021) in a study on 124 trios of SUDS cases. Moreover (Mutlu et al., 2015), reported a case of an infant born with skin and mucosal lesions and pyloric stenosis who died in the second month of life and had a different homozygous ITGB4 gene variant. Another study on deceased newborns in China identified different compound heterozygous variants in the ITGB4 gene in a newborn with epidermolysis bullosa (EB), metabolic acidosis, and disseminated intravascular coagulation (Yang et al., 2020). Furthermore (de Saint Basile et al., 2004), reported a CD3E variant in a child born to third-degree cousins, who later died. However, it was not clarified whether this diagnosis was made before or after the death of the child. ASPM variants were reported in a case of a stillbirth with microcephaly (Alamillo et al., 2016), a cohort of SIDS cases (Tester et al., 2018), and a cohort of fetal demise (So et al., 2022). No studies could be found on *postmortem* genetic analysis with the identification of MPV17, HEXB or TK2 gene variants. Still, MABP can be negative or may report benign variants only (Alghamdi et al., 2021). In the present study, a diagnosis was reached in 8 out of 11 tested individuals, with a diagnostic yield of 73%, highlighting the feasibility of MABP by ES in selected cases. Availability of biological samples from the proband is beneficial. In our series, biological samples from probands were available only in one case, where trio analysis was performed. Parental ES testing was carried out in the other 4 couples, leading to the identification of pathogenic variants and allowing a genotype-phenotype correlation. When a biological sample is unavailable, MABP can be performed (Alghamdi et al., 2021), like in the majority of our consultands. Anticipatory pre-mortem banking of a range of samples from children whose demise is expected can be helpful for reverse phenotyping, particularly where the proband exhibits an uncommon feature of the disorder (Sitaram et al., 2023). Moreover, the long-term storage of proband specimens through biobanks and other services may be beneficial but logistics and costs can be a hindrance (Deignan et al., 2023).

Consanguinity is known to contribute to genetic disorders, particularly autosomal recessive (AR) disorders (Shawky et al., 2013). It is prevalent and accepted in Middle Eastern communities, including Bahrain. However, over a span of 10 years, from 1990 to 2009, the rates of first-degree cousin marriages in Bahrain have experienced a notable decrease, declining from 24% to nearly 7% (Al-Arrayed and Hamamy, 2012). Four out of 6 couples in our cohort reported consanguinity (Alghamdi et al., 2021). Performed MABP (ES) in 83 consanguineous couples in Saudi Arabia, where consanguineous marriages are a common practice. In 43 families (52%), shared carrier status of 50 pathogenic/likely pathogenic variants was detected. Besides, 28 variants of uncertain clinical significance (VOUS) were detected in 24 families. Out of the couples examined, 10 couples shared the carrier status of several AR disorder-associated genes, which indicates a 12% risk of the offspring inheriting more than one genetic disorder. In our cohort, couple I exhibited a shared carrier status of two disorders, namely, CHS and MDDS. Based on our literature review, no similar cases were previously reported with these two genes in one family. Moreover, the mother exhibited compound heterozygosity of the LYST gene, but the other variant was assessed as VOUS. In another study from Saudi Arabia, more than one AR disorder was identified in 2.93% of the consanguineous families tested (3.54% for first-degree cousins or closer and 2.72% for second-degree cousins or further) (AlAbdi et al., 2021).

Consanguinity can be hidden for many generations, leading to high risk of very rare recessive disorders, especially in a small population like Bahrain. Two of our couples were non-consanguineous. In one of the couples, no final diagnosis was reached while in the other, the parents were heterozygous carriers of the same gene variant. Hidden or unexpected consanguinity is considered a challenge for homozygosity mapping (Miano et al., 2000; Woods et al., 2006). The topic of hidden consanguinity and its impact has been scarcely discussed in the literature otherwise. Hence avoiding consanguineous marriages might not be sufficient and the role of premarital genetic carrier testing cannot be emphasized enough.

Bereavement might contribute to late consultation and diagnosis. The couples in our series lost at least 2 years after the death of their children, prior to having the courage to seek other medical opinions, initiate investigations, and consider a new pregnancy. During the counseling sessions, all parents expressed their feelings of helplessness and guilt that accompanied the birth of a child suspected to have a rare genetic disease, which intensified with the child’s passing. Couple VI were beyond the window of conception at the time of presentation to the clinic but were just seeking peace of mind to understand if indeed an intervention could have been done earlier. After proper counseling and a thorough obstetrical evaluation, testing was not performed as it was determined that it would not provide any benefits to the couple. Bereaved parents are more likely to have depressive symptoms and marital disruption (Rogers et al., 2008).

Moreover, consultands may have poor adaptation to genetic information and results. Hence integration of psychological support and acknowledging their feelings of relief, loss, and disappointment is important at the time of counseling given all the above (Bates et al., 2019; Liang et al., 2022). Confirming a genetic cause of the child’s demise may provide grief support or, conversely, may accentuate the culpability felt by the parents (Chapple et al., 1995; James et al., 2006; Malek et al., 2019; Weaver et al., 2023). The details of these aspects were thoroughly discussed with all couples during the pre-testing counseling sessions. Couple VI expressed their relief of understanding the mechanisms of the disorder and the inheritance pattern but also the frustration of losing many years without a confirmatory diagnosis and the chance of having, in the fertile interval, a new pregnancy with the help of new technologies like preimplantation genetic testing for monogenic disorders (PGT-M). Investigations in such a case, on another note, could still prove beneficial for other family members at the reproductive age if in a consanguineous marriage.

Post-testing counseling, done in a comprehensive, nonjudgmental, and nondirective manner, is crucial for future pregnancy planning and outcomes. Identifying the pathogenic variants allowed the couples in our series to consider PGT-M in order to eliminate the risk of recurrence. PGT-M facilitates the detection of monogenic and chromosomal disorders (Parikh et al., 2021). This allows room for precision medicine in the subsequent management of the affected families (Shamseldin et al., 2018). Prenatal diagnosis is another option but raises important ethical issues. From the Islamic perspective, it is permissible to perform prenatal diagnosis if the risk to the embryo/fetus and the mother is minimal (A. Sultan et al., 2013; Shoaib, 2024). From our observation, the counseled couples did not favor prenatal diagnosis and opted directly for PGT-M.

Considering the relevant genes for the described phenotype, possible structural genomic rearrangements that are not highlighted by sequencing, targeting specific variants, and subsequently choosing the correct genetic test are essential to reach a diagnosis. No final diagnosis could be made in one couple (couple V). A biological sample was available from one of the two probands in this family but array, single gene sequencing, ES, and GS were negative. Suspected diagnoses in this couple included microdeletions or microduplications (del1q42, del6q or dup4q), Cockayne syndrome II, Pena-Shokeir syndrome II, and Schinzel-Giedion syndrome and none was confirmed by testing. In a meta-analysis, the diagnostic yield of GS for the detection of suspected genetic disorders among pediatric patients was 38.6% as compared to that of ES (37.8%). Mendelian disorders were better diagnosed (Nurchis et al., 2023). Re-analysis and RNA sequencing could be utilized where ES is negative or inconclusive, in conjugation with GS (Blake et al., 2023). Re-analysis of GS for couple V remained negative. Despite of all available technologies and increased coverage of GS, cases may remain unsolved even after an MABP, as complex genetic causes may be involved, beyond the testing capacities.

### Study limitations

Limitations of this study include the small number of subjects considering the clinic is a private facility which receives a limited number of patients as compared to government facilities. Additionally, the generally expensive cost of genetic testing is incurred by the families. Furthermore, many couples who have tragically lost their children to an undiagnosed disease may not be fully informed about additional genetic testing options that could aid and bring clarity prior to planning another pregnancy.

## Conclusion

Adequate parental genetic evaluation and counseling are mandatory to select the most appropriate genetic test that offers the greatest likelihood of confirming or clarifying the clinical diagnosis *postmortem*. We support the utilization of MABP due to the substantial diagnostic yield. A saved biological sample from the affected child or children will diminish the difficulty of identifying the genetic defect, decreasing the time and the cost of genetic testing, particularly in cases that lead to lethality. Hence a biobank of specimens from cases with no identified cause of death is a necessity. In the absence of biological samples from the affected or deceased children, conducting parental ES emerges as the optimal approach to identify the responsible genetic defects and enable subsequent appropriate management. Complete medical reports are very helpful in choosing the correct pipeline filters and interpret the tests results. In the delicate context of parental grief over their lost children, it is crucial to provide patience, empathy, and support to assist parents in comprehending complex genetic circumstances, making informed decisions, consenting to testing and future investigations, and coping with the results. It is imperative to encourage individuals and authorities to implement premarital and preconceptional genetic carrier testing, particularly in consanguineous populations and where testing is attainable.

## Data Availability

The original contributions presented in the study are included in the article, further inquiries can be directed to the corresponding author.
